# Supplementary material for: Content-rich biological network constructed by mining PubMed abstracts
Source: BMC Bioinformatics. 2004 Oct 8;5:147. doi: 10.1186/1471-2105-5-147 (PMC528731; doi:10.1186/1471-2105-5-147)
Supplement: Additional File 2 — The original results of the above study (non-essential files are deleted to keep the file size under the limit set by BMC bioinformatics). [file 1471-2105-5-147-S2.bz2 › chilibotAdditionalFile2/dip05/299ID7486683E1290/html/RASA1_PDGFRB.html]

 


 **RASA1** and **PDGFRB** 
  
Found 28 abstracts in PubMed, retrieved 05.  
 

 What does Google say? 
 PDF only 
| .edu only 

---

**Interactive relationship** (e.g. stimulation, inhibition, etc)

**Neutral relationship**- We have found that the F2 mutation has only a modest effect on ligand dependent activation of a chimeric PDGFR  [ **PDGFRB** ]  composed of the extracellular and transmembrane domains of the alphaPDGFR and the cytoplasmic domain of the betaPDGFR by three measures 1 the ability to phosphorylate endogenous and exogenous protein substrates in vitro, 2 phosphorylation of tyrosine 857, and 3 binding of the effector proteins PLCgamma, RasGAP  [ **RASA1** ] , and SHP 2.  Ref: 11281646 Exp Cell Res, 2001

**Non-interactive relationship** (e.g. studied together, co-existance, homology, etc.)

- Furthermore, we demonstrate that p62 dok can exert its negative effect on the PDGFR  [ **PDGFRB** ]  MAPK pathway independently of its ability to associate with RasGAP  [ **RASA1** ]  and Nck.  Ref: 11489946 J Exp Med, 2001
